# In Vivo Molecular MRI of ICAM-1 Expression on Endothelium and Leukocytes from Subacute to Chronic Stages After Experimental Stroke

**DOI:** 10.1007/s12975-017-0536-4

**Published:** 2017-05-16

**Authors:** Lisette H. Deddens, Geralda A.F. van Tilborg, Kajo van der Marel, Hedi Hunt, Annette van der Toorn, Max A. Viergever, Helga E. de Vries, Rick M. Dijkhuizen

**Affiliations:** 1 Biomedical MR Imaging and Spectroscopy Group, Center for Image Sciences, University Medical Center Utrecht, Utrecht, The Netherlands; 2 Department of Molecular Cell Biology and Immunology, VU University Medical Center, Amsterdam, The Netherlands

**Keywords:** Stroke, Inflammation, Intercellular adhesion molecule-1, Molecular imaging, Magnetic resonance imaging

## Abstract

Molecular MRI allows in vivo detection of vascular cell adhesion molecules expressed on inflamed endothelium, which enables detection of specific targets for anti-neuroinflammatory treatment. We explored to what extent MR contrast agent targeted to intercellular adhesion molecule-1 (ICAM-1) could detect endothelial- and leukocyte-associated ICAM-1 expression at different stages after experimental stroke. Furthermore, we assessed potential interfering effects of ICAM-1-targeted contrast agent on post-stroke lesion growth. Micron-sized particles of iron oxide (MPIO) functionalized with control IgG (IgG-MPIO) or anti-ICAM-1 antibody (αICAM-1-MPIO) were administrated at 1, 2, 3, 7, and 21 days after unilateral transient middle cerebral artery occlusion in mice, followed by in vivo MRI and postmortem immunohistochemistry. αICAM-1-MPIO induced significant contrast effects in the lesion core on post-stroke days 1, 2, and 3, and in the lesion borderzone and contralesional tissue on post-stroke day 2. αICAM-1-MPIO were confined to ICAM-1-positive vessels and occasionally co-localized with leukocytes. On post-stroke day 21, abundant leukocyte-associated αICAM-1-MPIO was immunohistochemically detected in the lesion core. However, MRI-based detection of αICAM-1-MPIO-labeled leukocytes was confounded by pre-contrast MRI hypointensities, presumably caused by phagocytosed blood remains. IgG-MPIO did not induce significant MRI contrast effects at 1 h after injection. Lesion development was not affected by injection of αICAM-1-MPIO or IgG-MPIO. αICAM-1-MPIO are suitable for in vivo MRI of ICAM-1 expression on vascular endothelium and leukocytes at different stages after stroke. Development of clinically applicable MPIO may offer unique opportunities for MRI-based diagnosis of neuroinflammation and identification of anti-inflammatory targets in acute stroke patients.

## Introduction

Molecular magnetic resonance imaging (MRI) enables detection of cellular and molecular processes in living organisms with the use of (super)paramagnetic contrast agents, with promising opportunities for in vivo studies on brain pathophysiology [1–3]. We and others have recently applied molecular MRI for the detection of neurovascular inflammation in rodent models of neurological disorders [4–7]. This has demonstrated the ability of molecular MRI to give distinctive insights into inflammatory events involved in cerebral pathology and to aid in monitoring the efficacy of anti-inflammatory treatment strategies in a longitudinal way, which may potentially lead to development of clinical protocols. Entities expressed on the luminal side of the cerebral endothelium that are induced by inflammation are particularly good targets for molecular MRI, because they can be easily reached by intravenously injected contrast agents. For instance, molecular MRI of enhanced P- and E-selectin expression after experimental brain injuries, e.g., stroke, has been demonstrated with the use of sialyl Lewis^X^ (sLe^X^)-targeted Gd-chelates [4] or iron oxides [6]. Similarly, vascular cell adhesion molecule-1 (VCAM-1) has been effectively targeted and imaged using antibody-functionalized iron oxides in a variety of brain disorders [5, 7].

We have recently developed an optimized molecular MRI approach for the detection of stroke-induced expression of intercellular adhesion molecule-1 (ICAM-1) [8]. ICAM-1 is a cell adhesion molecule which expression is increased during neurovascular inflammation and which is involved in the transmigration of leukocytes across the neurovasculature [9, 10]. ICAM-1 has been implicated in stroke pathophysiology and offers an effective therapeutic target for reduction of ischemic lesion development [11]. Experimental studies have demonstrated that injection of antibodies against ICAM-1 significantly reduce infarct size after transient unilateral stroke in rats [12]. Unfortunately, a clinical trial using a murine monoclonal anti-human ICAM-1 antibody (Enlimomab) did not show efficacy [13].

In contrast to the adhesion molecules P-selectin, E-selectin, and VCAM-1, ICAM-1 is not only expressed on inflamed endothelium, but also on activated leukocytes. This provides an interesting opportunity to image endothelial activation as well as leukocyte invasion in relation to stroke pathophysiology and (anti-inflammatory) treatment. However, it remains unknown to what extent molecular MRI of ICAM-1 expression in the brain reflects these specific inflammatory processes that may occur separately as well as jointly. The primary goal of our study was to determine the potential of ICAM-1-targeted MPIO for in vivo MRI of vascular ICAM-1 expression and leukocyte infiltration at different stages after transient unilateral stroke in mice. In addition, we assessed possible interfering effects of ICAM-1-targeted MPIO on post-stroke lesion development.

## Methods

### Preparation of Antibody-Functionalized MPIO

ProMag™ 1 Series, Bind-IT™ MPIO (25 mg MPIO/mL, 26.5% iron-content) were obtained from Bangs Laboratories, Inc. (Fishers, IN, USA) and extracted from their original buffer by magnetic separation and resuspended in coupling buffer (50 mmol/L 2-(*N*-morpholino)ethanesulfonic acid (MES); pH 5.2). Prior to the coupling procedure, a monoclonal antibody against mouse ICAM-1 (αICAM-1, YN1/1.7.4) and irrelevant immunoglobulin G antibody (IgG, RTK4530) (BioLegend; San Diego, CA, USA) were buffer-exchanged to coupling buffer by centrifugation, resulting in a final antibody concentration of 1.0 mg/mL. Next, MPIO and αICAM-1 or IgG were added in a 1:1 (*v*/*v*) ratio, vortexed and left to incubate for 60 min at room temperature on a roller-bench. Following incubation, antibody-MPIO were buffer-exchanged to storage solution (150 mmol/L NaCl, 0.002% azide) by magnetic separation and stored at 4 °C at a concentration of 12.5 mg MPIO/mL.

### Mouse Stroke Model

All animal procedures were approved by the Utrecht University Ethical Committee on Animal Experiments (protocol number: DEC 2011.I.10.097), and experiments were performed in accordance with the guidelines of the European Communities Council Directive. Eight-week-old C57Bl/6 mice, weighing 20 to 25 g (Harlan, Horst, The Netherlands) were anesthetized with isoflurane (3.5% induction, 1.5–2.0% maintenance) in air/O_2_ (2:1). Body temperature was maintained at 37.0 ± 0.5 °C. Transient focal cerebral ischemia was induced by 30 min right middle cerebral artery occlusion (MCAO) with an intraluminal filament [14]. In brief, a 7.0 polypropylene suture with a silicon-coated tip (tip diameter of 0.21 mm, Doccol Corporation, Redlands, CA, USA) was introduced into the right external carotid artery and advanced through the internal carotid artery until a slight resistance was felt, indicating that the MCA was occluded. During occlusion, the common carotid artery was clipped. After 30 min, the filament was withdrawn from the internal carotid artery and the clip was removed from the common carotid artery to allow full reperfusion. Before surgery, mice received a 1-mL subcutaneous (s.c.) injection of saline to compensate for loss of water and minerals, and a subcutaneous injection of 0.1 mg/kg buprenorphine (Temgesic; Schering-Plough, Houten, The Netherlands) for post-surgical analgesia.

### In Vivo MRI

Mice underwent in vivo MRI on a horizontal bore 9.4 T MR system (Agilent, Palo Alto, CA, USA), using an actively decoupled volume transmit coil (6 cm internal diameter; Rapid Biomedical, Rimpar, Germany) and a home-built surface receive coil (2.5 cm diameter). MRI was performed under general isoflurane anesthesia. Multi-slice multi-spin-echo MRI was applied for T_2_ mapping (repetition time (TR)/echo time (TE) 2300/12-96 ms; number of echoes (NE) 8; number of acquisitions (NA) 4; matrix size 192 (read-out (RO)) × 96 (phase-encoding (PE)); field-of-view (FOV) 20 × 20 mm^2^; 21 slices of 400 μm thickness; bandwidth 50 kHz; total scan duration 14 min and 43 s) to determine lesion size. T_2_*-weighted images (3D gradient echo; TR/TE 35/15 ms; NA 8; flip angle (FA) 10°; matrix size 160 (RO) × 96 (PE) × 80 (PE2); FOV 20 × 12 × 10 mm^3^; slab thickness 8 mm; 3 spatial saturation bands (8G/cm; 2 ms); bandwidth 50 kHz; 100 dummies; total scan duration 35 min and 54 s) were acquired before and up to 1 h after i.v. injection of IgG-MPIO or αICAM-1-MPIO (5 mg iron/kg body weight). Mice were sacrificed immediately after the last MRI scan by an overdose of isoflurane anesthesia followed by transcardial perfusion with PBS. Fresh brains were quickly excised, snap-frozen with liquid N_2_, and stored at −80 °C for postmortem immunohistochemistry.

### Postmortem Immunohistochemistry

For postmortem immunohistochemistry, cryosections of 10 μm (6 per mouse, ranging from approximately −1 to +1.5 mm from Bregma) were prepared. Subsequently, tissue was stained for either ICAM-1 or CD45, followed by Perls’ staining for MPIO, which could be easily detected (and differentiated from blood residues) because of their distinct large size and spherical shape. Therefore, tissue was dried overnight and aceton-fixated for 10 min at room temperature. After the aceton was evaporated, tissue was hydrated in PBS/0.1% BSA and incubated for 1 h with biotinylated αICAM-1 (10 μg antibody/mL of PBS/0.1% BSA; αICAM-1-biotin, YN1/1.7.4; BioLegend; San Diego, CA, USA) or biotinylated αCD45 (23 μg antibody/mL of PBS/0.1% BSA; αCD45-biotin, MP33; production in house), followed by incubation with horseradish peroxidase-labeled streptavidin (HRPstrep; DAKO, Glostrup, Denmark; prepared according to the manufacturer’s description) for 45 min. Diaminobenzidine solution (DAB; Sigma-Aldrich, St. Louis, MO, USA) was used as chromogen (used according to the manufacturer’s description). Tissue was subsequently incubated with Perls’ solution (2 mol/L HCl and 2% ferrocyanide in Milli-Q in 1:1 (*v*/*v*) ratio) for 20 min, thoroughly washed with Milli-Q, dipped in a nuclear fast red solution, dehydrated, and slides were mounted in Entallan.

### Experimental Protocol

#### Study I: Diagnostic Efficacy of αICAM-1-MPIO

To test the efficacy of αICAM-1-MPIO to detect post-stroke ICAM-1 expression on brain endothelium and/or infiltrated leukocytes, we conducted an explorative study in 53 mice that underwent transient MCAO followed by cross-sectional MRI at 1, 2, 3, 7, or 21 days. Six mice were excluded from the study: three mice died before MRI acquisition (day 1: *n* = 1; day 7: *n* = 2), two mice had no lesion on T_2_ maps (day 3: *n* = 1; day 21: *n* = 1) and in one mouse contrast agent was not properly injected (day 1: *n* = 1). After baseline MRI measurements, mice were injected with IgG-MPIO (day 1: *n* = 6; day 2: *n* = 5; day 3: *n* = 4; day 7: *n* = 4, day 21: *n* = 4) or αICAM-1-MPIO (day 1: *n* = 5; day 2: *n* = 5; day 3: *n* = 5; day 7: *n* = 4: day 21: *n* = 5) followed by post-contrast MRI.

#### Study II: Therapeutic Efficacy of αICAM-1-MPIO

To test the potential of αICAM-1-MPIO to affect lesion development, 24 mice underwent transient MCAO. Six mice died before MRI acquisition. A sample size of five to seven was used based on an earlier reported αICAM-1-induced lesion reduction of 40–45% after transient MCAO [12] (one-way ANOVA pairwise (two-sided equality); 20% standard deviation; *α* = 0.05; 1-*β* = 0.8). Treatment allocation was randomized. Absence of a stroke lesion on T_2_ maps at 1 day post-MCAO and unsuccessful injection of the contrast agent were used as exclusion criteria. Repeated MRI was done at day 1 and day 3 after MCAO. At day 1, directly after baseline MRI, mice received an i.v. injection of saline (*n* = 6; equal volume as MPIO injections), IgG-MPIO (*n* = 5), or αICAM-1-MPIO (*n* = 7). Follow-up MRI was performed 2 days after injection of saline, IgG-MPIO, or αICAM-1-MPIO.

### Image Analysis

For study I, brain lesions were identified by relatively high T_2_ values on T_2_ maps. Lesions were segmented, and homologous contralesional tissue was manually outlined on T_2_ maps for each animal. Perilesional tissue, i.e., the lesion borderzone, was defined by the expansion of the binary lesion segmentation obtained with a slice-by-slice twofold convolution with a 3 × 3 kernel, with exclusion from ventricles and corpus callosum. The lesional, perilesional, and contralesional segmentations were used as regions-of-interest (ROIs). To determine the amount of contrast agent accumulation within these ROIs, mean signal intensity (SI) on T_2_*-weighted images was measured at 30 min after MPIO injection (when free circulating MPIO should be largely cleared from the blood) for each animal, and expressed as absolute signal reduction according to (1).

1 $$ {\mathrm{SI}}_{\left(\mathrm{pre}\hbox{-} \mathrm{CA}\right)}{\hbox{-} \mathrm{SI}}_{\left(\mathrm{post}\hbox{-} \mathrm{CA}\right)} $$

To calculate the volume percentage of contrast-enhanced (CE) voxels within the ROIs, hypointense voxels with more than two standard deviations signal difference from pre-contrast T_2_*-weighted signal intensity, according to (2), were counted, and the volume of CE voxels was expressed as a percentage of total ROI volume, according to (3).

2 $$ {\mathrm{SI}}_{\left(\mathrm{CE}\ \mathrm{voxel}\right)}{<\mathrm{SI}}_{\left(\mathrm{ROI}\ \mathrm{pre}\hbox{-} \mathrm{CA}\right)}{\hbox{-} 2\mathrm{SD}}_{\left(\mathrm{ROI}\ \mathrm{pre}\hbox{-} \mathrm{CA}\right)} $$

3 $$ \left[\left(\mathrm{volume}\ \mathrm{of}\ \mathrm{CE}\ {\mathrm{voxels}}_{\left(\mathrm{post}\hbox{-} \mathrm{CA}\right)}\hbox{-} \mathrm{volume}\ \mathrm{of}\ \mathrm{CE}\ {\mathrm{voxels}}_{\left(\mathrm{pre}\hbox{-} \mathrm{CA}\right)}\right)/\mathrm{volume}\ \mathrm{of}\ \mathrm{ROI}\right]\times 100\% $$

The automated lesion segmentation procedure for study II consisted of a voxel-based supervised classification method that we have previously developed to identify stroke lesions [8]. First, two experts manually outlined lesions on T_2_ maps in ten animals. Based on the consensus between the expert segmentations, a random forest classifier was then trained on T_2_ and anatomical location obtained from non-rigid registration to a template image. The ipsilesional hemisphere was manually outlined on the T_2_ map by a blinded observer. Hemispheric lesion fraction was calculated according to (4).

4 $$ {\mathrm{volume}}_{\mathrm{lesion}}{/\mathrm{volume}}_{\mathrm{ipsilesional}\ \mathrm{hemisphere}}\times 100\% $$

### Statistical Analysis

Exclusion criteria for studies I and II were mortality before MRI, absence of a lesion in the MCA territory on T_2_ maps, or inadequate contrast agent delivery.

Linear mixed model analysis was performed using *R* software (R Development Core Team, 2011; https://www.r-project.org/; *nlme* and *multcomp* packages) to test for effects of ROI, contrast agent, time point, and their interactions in study I. For each ROI and time point, post hoc tests assessed the effects of contrast agent and the pairwise difference between the two contrast agents. *P* values were FDR-adjusted for multiple comparisons. Potential association between lesion volume and contrast-enhanced volume fraction was assessed with Pearson’s product moment correlation test. A one-way ANOVA was used to test for a treatment effect of αICAM-1-MPIO (i.e., significant reduction in hemispheric lesion fraction) in study II. *P* < 0.05 was considered significant. Values are presented as mean ± standard deviation.

## Results

### Lesion Development on T_2_ Maps

**Fig. 1:**
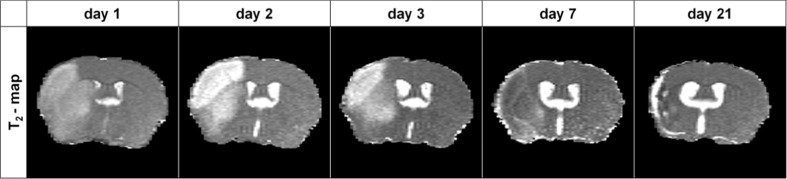
MRI of lesion development after stroke. Typical examples of unilateral lesions on T_2_ maps of a coronal mouse brain slice at different time points post-stroke. Lesioned tissue was characterized by T_2_ prolongation at days 1, 2, and 3, which partially (pseudo)normalized at day 7 and 21 (i.e., T_2_ “fogging”) along with tissue shrinkage

**Fig. 2:**
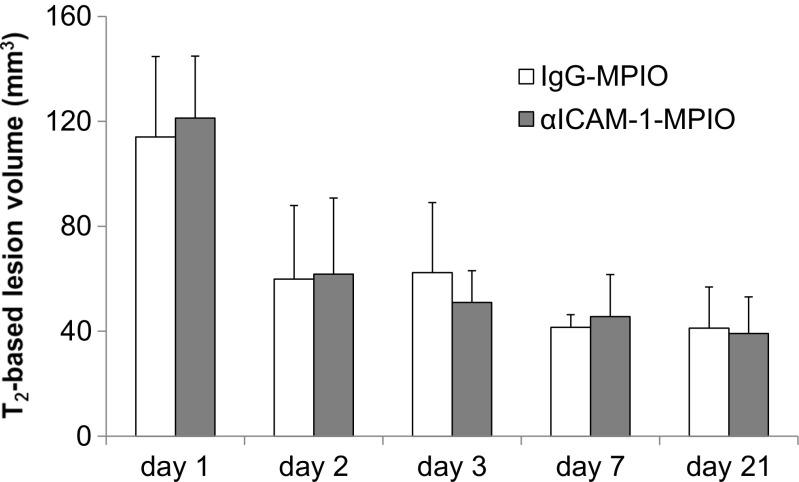
T_2_-based lesion volumes after stroke. Lesion volumes calculated from hyperintensity on T_2_ maps at days 1, 2, 3, 7, and 21 post-stroke. *Bars* represent mean + SD. There were no significant differences in lesion volume between mice allocated to the αICAM-1-MPIO or IgG-MPIO group at each time point

Transient MCAO induced unilateral lesions that were characterized by tissue T_2_ prolongation which was particularly evident at days 1, 2, and 3 post-stroke. T_2_ changes were more heterogeneous at days 7 and 21 post-stroke and accompanied by tissue shrinkage due to necrosis. Typical examples of T_2_ maps of coronal brain slices at different post-stroke time points are depicted in Fig. 1. Figure 2 shows the T_2_-based lesion volumes at different post-stroke time points before injection of IgG-MPIO or αICAM-1-MPIO.

### In Vivo T_2_*-Weighted MRI of αICAM-1-MPIO Binding at Different Time Points Post-Stroke

**Fig. 3:**
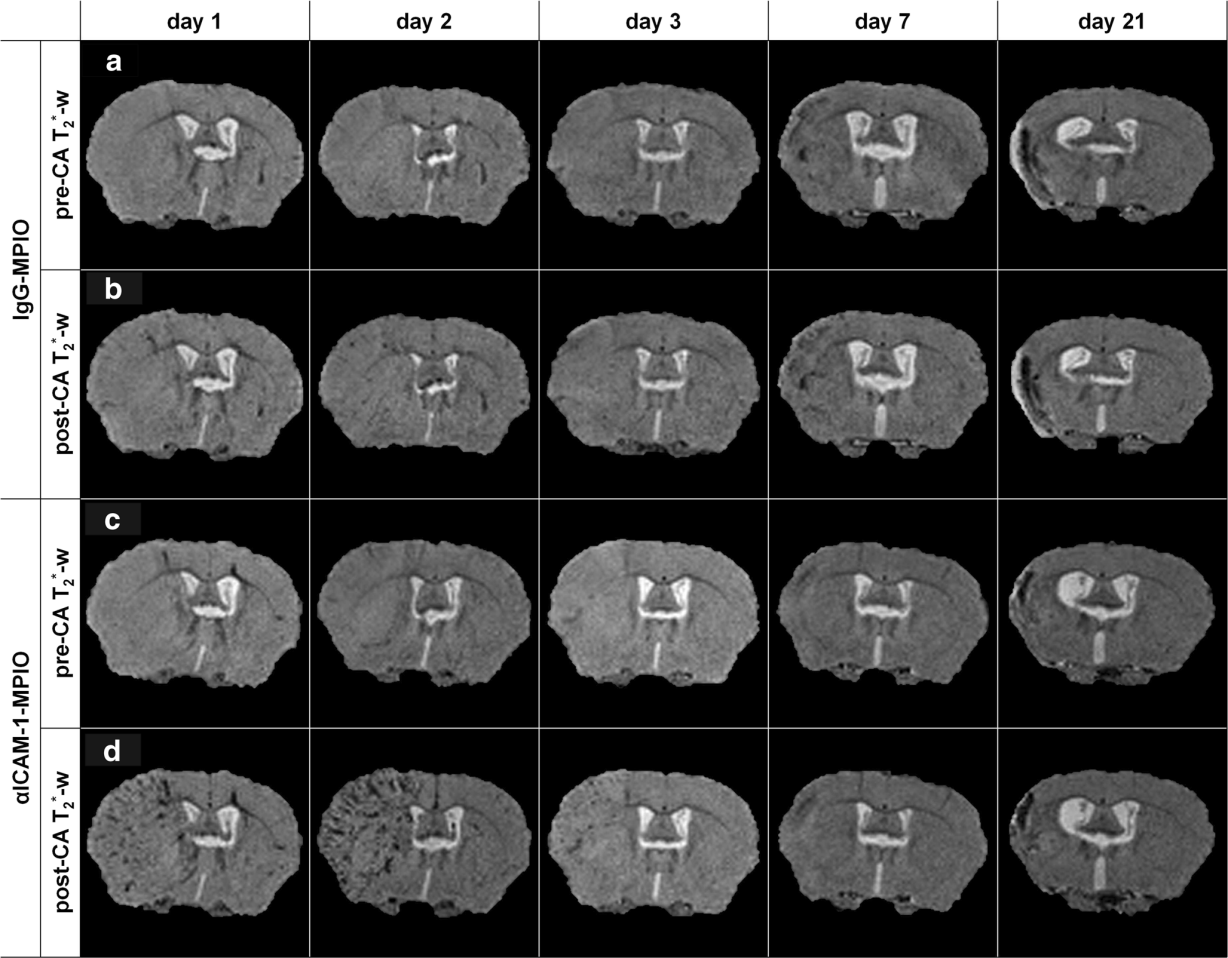
In vivo molecular MRI of ICAM-1 expression after stroke. In vivo molecular MRI displayed increased amounts of contrast-enhanced (hypointense) pixels in the lesion territory after injection of αICAM-1-MPIO, but not IgG-MPIO, at days 1, 2, and 3 post-stroke. Ipsilesional hypointense areas on pre-contrast agent (CA) T_2_*-weighted images were detectable at days 7 and 21, with no further signs of enhancement after contrast agent injection. **a**, **c** Pre-CA T_2_*-weighted (T_2_*-w) images. **b**, **d** Post-CA T_2_*-w images of a coronal brain slice of animals that received (**a**, **b**) IgG-MPIO or (**c**, **d**) αICAM-1-MPIO at 1, 2, 3, 7, or 21 days post-stroke

To test the efficacy of αICAM-1-MPIO to detect post-stroke ICAM-1 expression on brain endothelium and/or infiltrated leukocytes, cross-sectional MRI was done at 1, 2, 3, 7, or 21 days post-stroke. T_2_*-weighted images were acquired before and after MPIO injection. Typical examples of pre- and post-contrast T_2_*-weighted images are shown in Fig. 3. In the pre-contrast T_2_*-weighted images (Fig. 3a, c), relatively large hypointense regions were discernible in the ipsilesional hemisphere at 7 and 21 days post-stroke, which was not evident at post-stroke days 1, 2, and 3. Scarce signal reductions could be seen throughout the entire brain after injection of IgG-MPIO at all time points (Fig. 3b). In contrast, substantial post-contrast hypointensities were observed after injection of αICAM-1-MPIO at day 1, day 2 and, to lesser extent, at day 3 post-stroke (Fig. 3d). These hypointensities were largely confined to the ipsilesional hemisphere and displayed a typical macroscopical pattern that suggest their association with cerebral vessels. Contrast enhancement was absent or minimal when αICAM-1-MPIO were injected at 7 days post-stroke. At day 21, pre-contrast hypointense regions enlarged after αICAM-1-MPIO injection, however, without an apparent vascular pattern as seen at days 1, 2, and 3.

**Fig. 4:**
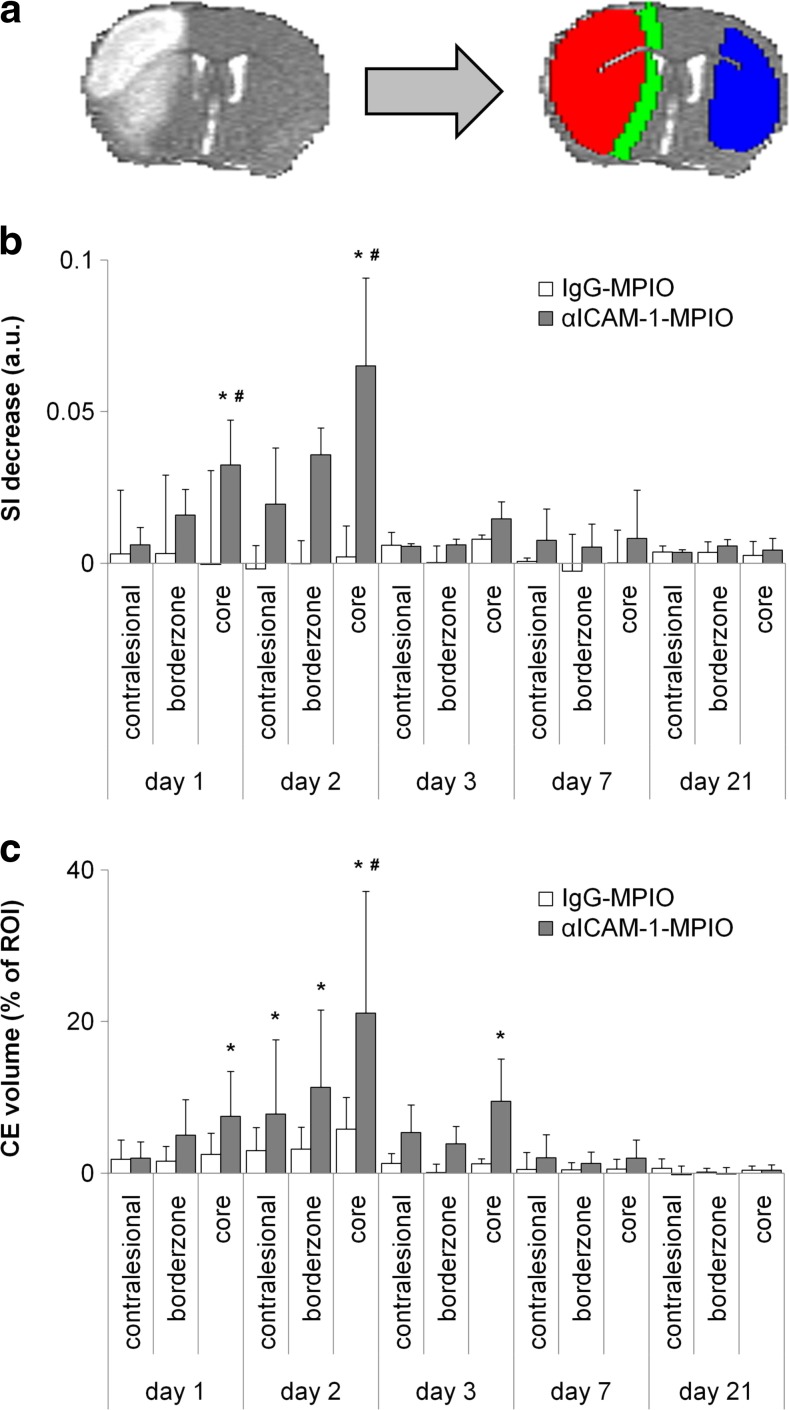
MRI-based measurement of regional expression of ICAM-1 after stroke. αICAM-1-MPIO induced significant T_2_*-weighted signal intensity reduction and increased contrast-enhanced volume subacutely after stroke. **a** Typical example of ROI selection based on lesion identification on a T_2_ map (*left*), shown with lesion core (*red*), i.e., tissue area with clearly elevated T_2_, lesion borderzone (*green*), i.e., defined by expansion of the lesion core with a slice-by-slice two-fold convolution with a 3 × 3 kernel, with exclusion from ventricles and corpus callosum, and contralesional ROI (*blue*) as overlays (*right*). Bar graphs showing **b** absolute decrease in T_2_*-weighted signal intensity (*SI*), and **c** contrast-enhanced (*CE*) volume, i.e., increase in amount of hypointense voxels, in contralesional, borderzone, and core ROIs after injection of IgG-MPIO or αICAM-1-MPIO at different time points after stroke. *Bars* represent mean + SD. *P < 0.05 compared to pre-injection, #*P* < 0.05 compared to IgG-MPIO

For quantitative image analysis, contrast-induced T_2_*-weighted signal intensity decrease and volume percentage of contrast-induced hypointensities were calculated in ROIs, i.e., lesion core, lesion borderzone, and contralesional homologous tissue (Fig. 4a). T_2_*-weighted signal intensity after administration of αICAM-1-MPIO clearly decreased in the ipsilesional hemisphere at post-stroke days 1 and 2, which was statistically significant in the lesion core (day 1: *P* = 0.016; day 2: *P* < 0.001), as compared to post-IgG-MPIO injection. Post-contrast signal decreases were not significant at days 3, 7, and 21 post-stroke.

Significant increase in the volume percentage of hypointense voxels was observed in the lesion core at days 1, 2, and 3 post-stroke after αICAM-1-MPIO injection (7.5 ± 5.9, 21.1 ± 16.1, 9.5 ± 5.6%, respectively). Significantly enlarged volumes of contrast enhancement were also measured in the lesion borderzone (11.3 ± 10.2%) and contralesional tissue (7.8 ± 9.8%) at post-stroke day 2. No significant MPIO-induced enlargement of hypointense volume was detected at day 7 or day 21 post-stroke. There was no significant correlation between lesion volume and αICAM-1-MPIO-enhanced volume fraction in the lesion core at day 1 post-stroke (*R* = 0.73; 95% confidence interval: −0.44 to 0.98; *P* = 0.17).

### Immunohistochemical Detection of MPIO in Postmortem Brain Tissue

**Fig. 5:**
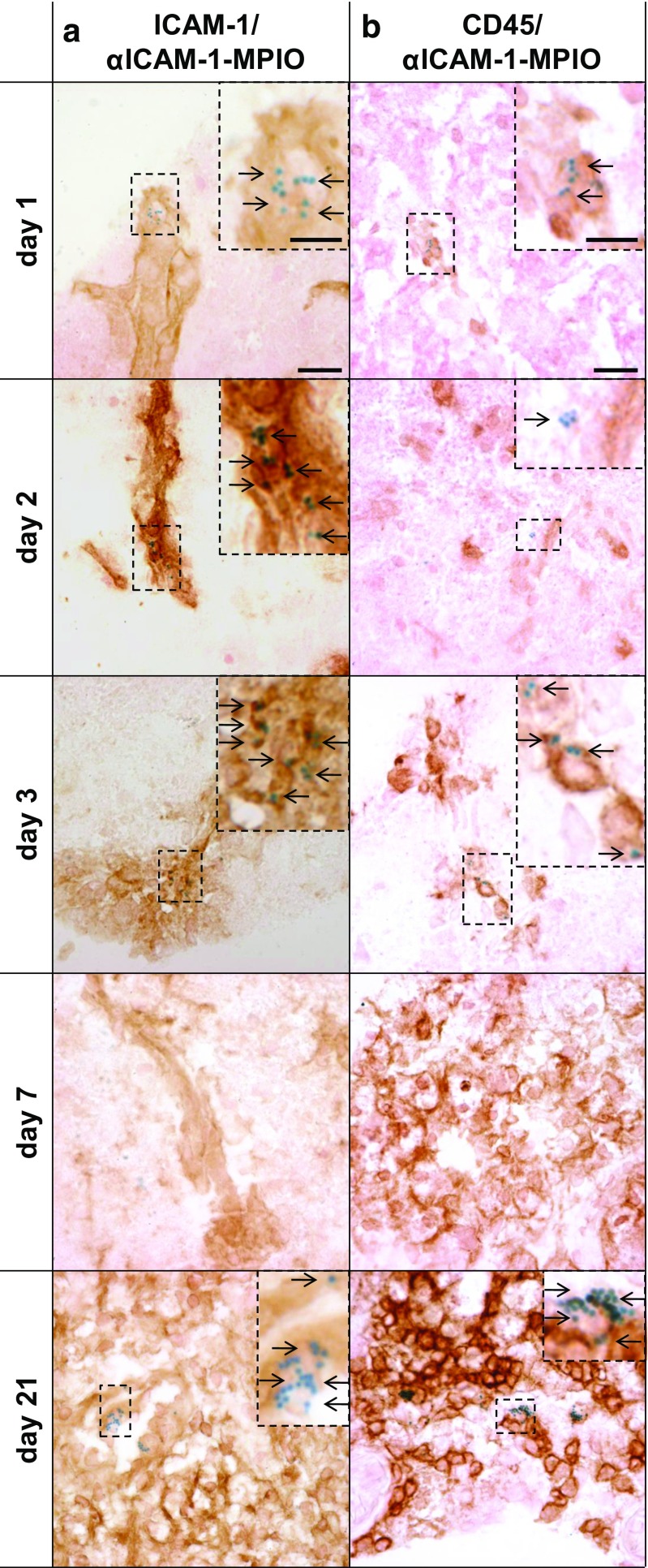
Immunohistochemical confirmation of αICAM-1-MPIO binding. Immunohistochemical analysis of the sensorimotor cortex in the lesion territory revealed that αICAM-1-MPIO were confined to ICAM-1-positive vessels, and occasionally co-localized with leukocytes at post-stroke days 1, 2, and 3, but were abundantly associated with leukocytes at day 21. MPIO presence was undetectable at day 7. **a** ICAM-1 staining (*brown*) and MPIO presence (*blue*), and **b** CD45 staining (*brown*) and MPIO presence (*blue*), with magnified inserts showing ipsilesional cortical tissue containing CD45- and MPIO-positive cells. *Arrows* indicate MPIO presence. *Scale bars*, shown in the images obtained at day 1, represent 25 μm in the overview image and 10 μm in the inserts

After MRI, postmortem brain tissue was immunohistochemically analyzed for the presence of ICAM-1, leukocytes (CD45), and MPIO. Light microscopy images of ipsilesional cortical brain tissue of mice that received αICAM-1-MPIO are shown in Fig. 5. At post-stroke days 1 and 2, MPIO were abundantly present in the lesion area and strictly confined to ICAM-1-positive structures which displayed vascular morphology. Occasionally, co-localization of MPIO with CD45-positive leukocytes was observed. At post-stroke day 3, detected MPIO were not only present at the ICAM-1-positive vasculature but also co-localized with vasculature-confined CD45-positive leukocytes. However, the amount of ICAM-1-positive vessel-like structures as well as MPIO presence were reduced at this stage compared to the earlier post-stroke phases, which is in agreement with the MRI findings. At day 7, MPIO were hardly detected within the brains of analyzed animals. At day 21, massive leukocyte infiltration was apparent. ICAM-1 staining was clear, but without a typical vascular pattern as seen at post-stroke days 1, 2, and 3. Considerable amounts of MPIO were found throughout the lesion core, associated with leukocytes. Tissue from animals that received IgG-MPIO revealed no MPIO presence at different time points (data not shown).

### Effect of αICAM-1-MPIO on Lesion Development

Despite effective vascular binding in the lesion area at day 1 after stroke, as demonstrated with in vivo MRI and postmortem immunohistochemistry, αICAM-1-MPIO did not significantly influence lesion size. Animals that received αICAM-1-MPIO at day 1 displayed similar change in hemispheric lesion fraction between day 1 and day 3 (−7 ± 29%), as compared to animals that received IgG-MPIO (−1 ± 29%) or saline (−2 ± 13%).

## Discussion

This study aimed to determine the diagnostic potential of ICAM-1-targeted MPIO for in vivo MRI of vascular ICAM-1 expression and leukocyte infiltration at different stages after stroke in mice. To this end, IgG-MPIO or αICAM-1-MPIO were injected at 1, 2, 3, 7, and 21 days after transient unilateral MCA occlusion. In addition, we assessed whether αICAM-1-MPIO injection at post-stroke day 1 exerted a possible therapeutic effect by limiting post-stroke lesion development.

We showed that αICAM-1-MPIO binding was clearly detectable with in vivo MRI at days 1, 2, and 3 post-stroke in the lesion core, and at day 2 also in the borderzone of the lesion and contralesional tissue. Immunohistochemistry on postmortem brain tissue showed that the αICAM-1-MPIO accumulation at these subacute post-stroke stages was confined to ICAM-1-positive vessel-like structures, and occasionally co-localized with vessel-restricted leukocytes. Normalization of baseline (i.e., before contrast agent injection) T_2_ values at day 7 reflect the so-called “fogging effect” [15], in which MRI signal intensity in ischemic areas transiently normalizes, which may be explained by resolution of edema and/or inflammatory responses [16]. Indeed, this was histologically confirmed by the increased amount of CD45-positive cells that we detected at this stage. At day 21, MRI was not able to depict the immunohistochemically detected αICAM-1-MPIO presence, which was mainly co-localized with leukocytes. We speculate that αICAM-1-MPIO bound to circulating leukocytes in the blood at this chronic post-stroke stage, followed by migration into the lesioned tissue. Alternatively, αICAM-1-MPIO may have accumulated in the lesion at sites of extensive damage to the blood-brain barrier, followed by engulfment by activated microglia or infiltrated leukocytes. However, IgG-MPIO were not detected in brain tissue at any time point with MRI or immunohistochemistry, which makes the latter explanation less likely. Despite effective vascular binding, no therapeutic effect of αICAM-1-MPIO compared to saline or IgG-MPIO injection was measured in this study.

Our study describes two different methods to quantify the presence of contrast agent from T_2_*-weighted MR images: (i) contrast-induced decrease in signal intensity and (ii) increase in volume of contrast-enhanced voxels. Our analyses showed that both approaches rendered comparable results. Nevertheless, the volumetric analysis revealed more statistically significant differences between targeted and non-targeted contrast agents. Ideally, a voxel-by-voxel analysis of pre- and post-contrast images should be performed. However, this was unfeasible due to slight movements of the brain during MRI acquisitions.

The highest degree of αICAM-1-MPIO presence at day 2 in our study corresponds with previous histological studies that reported maximal ICAM-1 expression at this stage after transient MCAO in rodents [8, 17, 18]. MPIO presence was confined to ICAM-1-positive vessels, which occasionally co-localized with leukocytes in the vascular space at post-stroke days 1, 2, and 3. We detected no signs of MPIO associated with leukocytes in the extravascular space, which suggests that αICAM-1-MPIO was only linked to blood-borne leukocytes that had not extravasated into the brain parenchyma but retained on the luminal side of the vasculature. At 21 days after stroke, CD45 staining suggested massive infiltration of leukocytes, although it may have also partly reflected activated proliferating microglia in lesioned tissue. αICAM-1-MPIO were also abundantly present, but this was not distinctly detected with MRI. The lack of contrast enhancement may likely be explained by the very short T_2_ and T_2_* values as is evident from strong hypointensities on T_2_ maps and T_2_*-weighed images before injection of contrast agent. This pre-contrast loss of MRI signal in the lesion area hampers efficient detection of subsequent (signal reducing) MPIO accumulation. The pre-contrast hypointense region, preceded at day 7 by T_2_ (pseudo)normalization or “fogging”, corresponded with the area of massive cell infiltration and/or activated proliferating microglia activation as identified with immunohistochemistry. The MR signal reduction may be explained by high iron content due to phagocytosis of blood remains [19]. Similarly, in a previous study, the presence of iron-containing inflammatory cells hampered the detection of engrafted superparamagnetic iron oxide (SPIO)-labeled stem cells in a photothrombotic stroke model in rats [20].

Our findings suggest that αICAM-1-MPIO may also be used for cellular MRI purposes, which typically involve in vivo or in vitro cellular incorporation of non-targeted contrast agent [1]. However, it should be noted that MPIO may not permanently label cells, as dissociation from their target has been previously reported [7, 8]. To explore the use of αICAM-1-MPIO for cellular imaging, it would be of interest to determine the contribution of cell-associated MPIO to the observed MR contrast enhancement by depletion of systemic leukocytes prior to αICAM-1-MPIO administration [21]. Furthermore, future studies should also look into possible differences in ICAM-1-MPIO binding between specific leukocyte subsets. In this study, we only employed staining of a general leukocyte marker (CD45) to determine co-localization of MPIO with any type of leukocyte.

Our data also indicate that αICAM-1-MPIO does not affect lesion development in a similar way as was found with anti-ICAM-1 treatment by Zhang and co-workers [12]. For our molecular MRI study, we used 5 mg iron per kg body weight to induce efficient MR contrast with αICAM-1-MPIO, which corresponded with 0.75 mg anti-ICAM-1 monoclonal antibody. Zhang and co-workers have shown that a dose of 2 mg anti-ICAM-1 monoclonal antibody per kg body weight infused 1 h after reperfusion in a rat MCA occlusion model, followed by an extra dose of 1 mg antibody per kg body weight at 22 h after reperfusion, significantly decreased lesion volume with 41%. The lower, single dose and later administration of antibody, but also a larger variation in lesion size, in our study may explain lack of a similar therapeutic effect. Furthermore, antibodies conjugated to MPIO are presented in a multivalent way to increase target efficacy at a relatively low dose of injected particles. This is an optimal particle design for diagnostic purposes, but it may restrict therapeutic potency.

To conclude, this study shows that molecular MRI with αICAM-1-MPIO offers a unique approach for in vivo imaging of endothelial ICAM-1 expression and vascular leukocyte adhesion after experimental stroke, without significantly affecting lesion development. Recent development of clinically viable biodegradable MPIO [22] may open the door to future diagnostic applications in stroke patients, in which early detection of endothelial activation may guide (anti-inflammatory) treatment decision-making.
